# Disrupted Functional Brain Network Topology in Etomidate Misuse

**DOI:** 10.31083/AP49872

**Published:** 2026-06-25

**Authors:** Ying Tang, Xingmin Wang, Juan Le, Qiuping Huang, Hao Chen, Xinxin Chen, Xuhao Wang, Yunde Tang, Lishun Zhao, Lin Zhao, Hongxian Shen, Zhenjiang Liao

**Affiliations:** ^1^Department of Psychiatry, National Clinical Research Center for Mental Disorders, The Second Xiangya Hospital of Central South University, 410011 Changsha, Hunan, China; ^2^Department of Psychology, School of Humanities and Management, Hunan University of Chinese Medicine, 410208 Changsha, Hunan, China; ^3^Hunan Lituo Compulsory Isolation and Drug Rehabilitation Center, Hunan Lituo Drug Rehabilitation and Recovery Center, 410113 Changsha, Hunan, China; ^4^Hunan Provincial Lushan Compulsory Isolation Detoxification Center, 410000 Changsha, Hunan, China

**Keywords:** etomidate, substance-related disorders, brain mapping, resting state functional magnetic resonance imaging, addiction, machine learning

## Abstract

**Background:**

Etomidate misuse (EM) has recently become an increasing public health concern in East and Southeast Asia, but its neurobiological mechanisms are still not well understood. Although substance use disorders (SUDs) are commonly associated with disruptions in large-scale brain network organization, the effects of EM on brain network topology remain largely unexplored.

**Methods:**

Resting-state functional magnetic resonance imaging (rs-fMRI) data were acquired from individuals with EM and healthy controls (HC). Graph theoretical analyses were employed to and characterize global and nodal topological properties of functional brain networks. Clinical assessments captured substance use characteristics, craving, impulsivity, and addiction severity. Partial correlation analyses were conducted to examine associations between network metrics and substance use characteristics. Additonally, a support vector machine (SVM) classifier was implemented to discriminate individuals with EM from HC based on network features.

**Results:**

A total of 103 individuals with EM and 57 HC were included in the final analysis. Global topological organization that appeared was largely preserved in the EM group, with the exception of a significantly reduced clustering coefficient. At the nodal level, individuals with EM exhibited significant alterations in degree centrality, betweenness centrality, and nodal efficiency across regions predominantly distributed within the default mode, attention, and sensorimotor networks. Correlation analyses revealed no significant associations between network metrics and substance use characteristics following correction for multiple comparisons. Furthermore, the SVM model achieved moderate classification performance (accuracy = 66.7%) with an area under the curve (AUC) of 0.711.

**Conclusions:**

This study provides the first systematic investigation of the brain network topology in EM. The findings indicate widespread alterations in nodal network properties alongside relatively preserved global topological organization. While these results may offer preliminary indicators related to EM, their clinical relevance requires further in future research.

## Main Points

1. Individuals with etomidate misuse (EM) exhibit significant abnormalities in global topological properties and more extensive changes in nodal properties across multiple brain regions, particularly within the default mode network, attention networks, and sensorimotor areas.

2. A support vector machine (SVM) model based on network metrics provided preliminary classification performance (66.7%) in distinguishing EM individuals from healthy controls (HC), highlighting the potential of brain network features as diagnostic tools.

3. This study is the first to investigate brain network topology in EM, advancing our understanding of its neurobiological basis and offering insights for diagnosis and treatment monitoring.

## 1. Introduction

Etomidate is an ultrashort‐acting nonbarbiturate sedative that has recently become a drug of misuse, particularly in East and Southeast Asia [1]. The number of people who use etomidate problematically has grown rapidly. For instance, data from 2024 showed that over 33,000 individuals in China were engaged in problematic use of etomidate, marking it as an emerging public health crisis [2]. Clinical and toxicological case series have demonstrated that chronic problematic etomidate use is associated not only with acute neurological symptoms such as myoclonus and dissociation, but also with serious endocrine consequences including adrenal suppression and cortisol dysregulation. Furthermore, individuals with chronic problematic etomidate use frequently present with mood disorders, hallucinations, delusions, heightened impulsivity, and features consistent with substance use disorder [1,3,4,5]. Although there is growing evidence of its addictive potential and increasing prevalence, the neurobiological mechanisms underlying etomidate misuse (EM) remain largely unknown. This knowledge gap presents a significant challenge in developing effective interventions and diagnostic tools for EM.

Substance use disorder (SUD) is a chronic, relapsing brain disease. Its pathogenesis involves dysfunction across multiple large-scale neural networks, with core manifestations that include reward processing dysregulation, emotional dysregulation, and impaired cognitive control [6]. Understanding SUD from a network perspective is key, as addiction results not from isolated brain region abnormalities but from disrupted communication across brain systems [7,8]. Resting-state functional magnetic resonance imaging (rs-fMRI) combined with graph theory analysis has become a valuable tool for studying brain network organization [9]. This approach models the brain as a complex network of functionally interacting nodes (brain regions, neurons, or voxels) connected by edges (functional relationships), enabling precise quantification of network topology and efficiency [10]. Critically, graph theory metrics can capture both global network properties (e.g., integration and segregation) and regional nodal characteristics (e.g., centrality and efficiency), providing a comprehensive framework for understanding brain network dysfunction in SUD.

Graph theory neuroimaging studies suggest that different substances associated with problematic use produce distinct patterns of brain network topological abnormalities. For instance, individuals with methamphetamine use disorder exhibit reduced small-world characteristics and modularity in white matter networks, alongside elevated nodal efficiency in the right superior temporal gyrus, globus pallidus, and ventromedial prefrontal cortex—regions primarily within the default mode network [11,12]. In contrast, a whole-brain resting-state graph-theoretical analysis demonstrated that recent cocaine use was associated with decreased efficiency in fronto-temporal and subcortical networks, predominantly encompassing the salience, semantic, and basal ganglia networks, alongside increased efficiency in the visual network [13]. Additionally, individuals with cannabis use disorder show significantly reduced global network efficiency alongside increased clustering coefficients, with altered local network organization particularly evident in the cingulate cortex [14]. Collectively, these findings suggest that specific substances may produce signature patterns of network disruption, likely reflecting their unique pharmacological mechanisms and the particular neural circuits they target. Given etomidate’s distinct pharmacological profile as a positive allosteric modulator of Gamma-Aminobutyric Acid Type A (GABA_A) receptors with preferential binding to β2/β3-containing receptors, it is plausible that EM may be associated with a unique pattern of brain network alterations that differs from those observed in other substance misuse conditions [15,16].

However, the brain network topology of EM has never been systematically investigated. Existing research on etomidate has predominantly focused on its anesthetic properties [17,18,19], with limited attention to its addictive potential and the neural substrates of problematic use. Consequently, whether EM is characterized by distinct alterations in brain network topology, and whether such alterations correlate with clinical features remains unexplored. Addressing this gap is essential not only for understanding the neurobiology of EM but also for identifying potential neuroimaging biomarkers that could inform clinical diagnosis and treatment monitoring. To fill this knowledge gap, we used graph theory methods to analyze the topological properties of resting-state functional brain networks in individuals with EM. By comparing global and nodal graph theory metrics between EM individuals and healthy controls (HC), we aimed to: (1) identify topological property abnormalities in brain regions and networks associated with etomidate misuse; (2) examine correlations between network alterations and substance use characteristics; and (3) evaluate the potential of network-based features as diagnostic biomarkers using support vector machine (SVM) classification. To our knowledge, this is the first neuroimaging study to investigate functional brain network topology in EM.

Based on prior findings in other substance use disorders and etomidate’s unique pharmacological profile, we formulated the following hypotheses: (1) EM is associated with significant alterations in the topological properties of functional brain networks, potentially affecting node properties; (2) The degree of functional brain network disruption in EM correlates with clinical features of addiction; (3) Machine learning models using network topology metrics can provide preliminary classification performance in distinguishing individuals with EM from healthy controls, highlighting the potential of these features as neuroimaging biomarkers.

## 2. Materials and Methods

### 2.1 Participants

This study recruited individuals with EM from Hunan Province between 2024 and 2025 through cooperation with local treatment facilities. Recruitment was based on a convenience sampling approach, including all eligible participants who met the inclusion and exclusion criteria during the recruitment period.

The inclusion criteria for the EM group were as follows: (1) meeting Diagnostic and Statistical Manual of Mental Disorders, Fifth Edition (DSM-5) diagnostic criteria for substance use disorder, with etomidate identified as the primary substance involved [20]; (2) having used etomidate at least twice per month over the past year, continuously for three months or more; (3) aged between 16 and 40 years, male, right-handed, and with normal or corrected-to-normal vision; (4) a standard score of ≥70 on Raven’s Progressive Matrices [21,22]. The exclusion criteria were as follows: (1) contraindications to MRI scanning; (2) prior regular medication or psychological therapy; (3) presence of severe physical illnesses or major medical conditions requiring regular medication; (4) positive screening for major psychiatric disorders using the Mini International Neuropsychiatric Interview (MINI) (e.g., psychotic disorders, bipolar disorder) [23,24]; (5) a family history of mental disorders; (6) non-nicotine substance use disorders; (7) diagnosis of behavioral addictions, including gambling disorder and internet gaming disorder, as assessed during clinical interviews.

The HC group was recruited from the local community through advertisements, including online ads and posters, and through social media platforms such as Weibo, WeChat, and QQ. The inclusion criteria for the HC group were: ethnicity and age-matched healthy males with no history of psychiatric disorders or substance use disorders, with the exception of tobacco use. Given that EM in this study population predominantly occurs via electronic cigarette devices, nicotine use disorder was highly prevalent among participants with EM and was allowed in both study groups. All participants were assessed for eligibility by trained clinicians with addiction medicine expertise.

### 2.2 Procedure

All participants underwent a structured clinical assessment using the MINI. Participants with EM underwent 3.0T brain MRI scanning and clinical assessment, including evaluations of substance use patterns as well as factors related to craving and impulse control. All participants were scanned during a period of abstinence. No participants were receiving psychotropic medications, opioid substitution therapy, or neuromodulation interventions (e.g., repetitive transcranial magnetic stimulation (rTMS), deep brain stimulation (DBS)) at the time of scanning, and none were in an acute withdrawal state during scanning.

### 2.3 Measures

#### 2.3.1 Patterns of Substance Use

Clinical assessments were conducted by qualified psychiatrists using structured interviews based on the DSM-5 criteria for substance use disorder. The assessment included 11 diagnostic items, with each item scored as “1” for a positive response or “0” for a negative response. Each participant completed a questionnaire that included demographic information, age at first use, cumulative use duration (months) and average single-use dose (g), in order to evaluate patterns of substance use.

#### 2.3.2 Craving

The Desire for Drug Questionnaire (DDQ), was employed to assess participants’ substance craving [25]. All 13 items use a 7-point Likert scale. The scale has demonstrated good reliability and validity in studies involving various addictive substances across multiple countries (Cronbach’s α = 0.86) [26].

#### 2.3.3 Impulsivity

The Chinese version of the Barratt Impulsiveness Scale, 11th Edition (BIS-11) was used to assess participants’ impulsive traits. A 4-point Likert scale was used (1 = Almost Never, 4 = Almost Always), with higher scores indicating more prominent impulsive traits. The scale has shown good reliability and validity in substance-dependent populations, with a Cronbach’s α coefficient of 0.79–0.83 [27,28,29].

### 2.4 MRI Data Acquisition and Preprocessing

This study utilized a 3.0T GE SIGNA Architect MRI scanner (GE Medical Systems, LLC, Waukesha, WI, USA; https://www.gehealthcare.com/en-us/products/magnetic-resonance-imaging) for data collection. Specific scanning parameters were as follows: rs-fMRI: repetition time (TR) = 1000 ms, echo time (TE) = 30 ms, number of slices = 40, flip angle = 90°, field of view (FOV) = 220 × 220 mm, matrix = 64 × 64, slice thickness = 3.6 mm, slice gap = 0 mm, voxel size = 3.4 × 3.4 × 3.6 mm. T1-weighted images (used for functional image registration): TR/TE = 7.4 ms/2.2 ms, number of slices = 188, matrix size = 256 × 256, flip angle = 10°, FOV = 256 × 256 mm, slice thickness = 1 mm, slice gap = 0 mm, voxel size = 1.0 × 2.6 × 1.0 mm. Participants were asked to remain still, close their eyes, and stay awake.

Functional MRI data preprocessing was performed using the GRETNA toolbox version 2.0.0 (National Key Laboratory of Cognitive Neuroscience and Learning, Beijing Normal University, Beijing, China; https://www.nitrc.org/projects/gretna) within MATLAB R2018b (The MathWorks, Inc., Natick, MA, USA; https://www.mathworks.com) [30], which included the following steps: (1) removal of the first 10 time points to mitigate initial magnetic field instability; (2) slice timing and head motion correction, excluding subjects with displacement >2 mm or rotation >2° and mean framewise displacement (mean FD) was calculated [31]; (3) co-registration of EPI images to individual T1-weighted images and spatial normalization to the Montreal Neurological Institute (MNI) space with a resampled voxel size of 3 mm × 3 mm × 3 mm; (4) temporal band-pass filtering (0.01–0.08 Hz); and (5) nuisance regression of white matter and cerebrospinal fluid signals, followed by linear detrending.

### 2.5 Network Construction and Analysis

First, we employed the Dosenbach atlas to parcellate the functional images into 160 regions of interest (ROIs), which served as network nodes [32]. For each participant, a 160 × 160 functional connectivity matrix was constructed by extracting the time series from each parcellation unit and computing pairwise functional connectivity using Pearson correlation coefficients. Based on the connectivity matrix, a weighted network graph was generated for each subject, incorporating only positive correlations above a predefined threshold.

Sparsity was used to assess brain network connection density. The lower bound of sparsity was determined using the GRETNA gretna_get_rmax function, which was calculated to be 0.032; a threshold of 0.03 was then applied for consistency across all subjects. The upper bound was set to 40% (with 1% intervals) based on prior literature [33,34]. We computed the following global graph-theoretical metrics: small-worldness (σ), global efficiency (E_g_), local efficiency (E_loc_), clustering coefficient (C_p_), and shortest path length (L_p_). The area under the curve (AUC) was calculated for each of these global metrics across the sparsity range for subsequent statistical analysis. In addition, nodal metrics were evaluated, including degree centrality, betweenness centrality, nodal efficiency, nodal local efficiency, shortest path length, and nodal clustering coefficient.

### 2.6 Statistical Analysis

IBM SPSS Statistics 27.0 (IBM Corp., Armonk, NY, USA; https://www.ibm.com/support/pages/downloading-ibm-spss-statistics-27) was used for statistical analyses. Continuous variables were tested for normality using the Shapiro–Wilk test. Normally distributed data are presented as mean ± standard deviation (M ± SD), while non-normally distributed data are presented as median (P25, P75). Categorical variables are presented as frequencies (percentages) [n (%)]. For group comparisons, demographic and clinical variables between the EM and HC groups were analyzed using *t*-tests, Mann-Whitney U tests, or chi-square tests, depending on the data type. Substance use characteristics specific to the EM group (e.g., craving, dosage, withdrawal duration) were described descriptively.

For each global graph theory metric, differences in AUC between the EM and HC were compared. Node-metric pairs showing significant inter-group differences after multiple-comparison correction were retained for follow-up analysis. Within the EM group, partial correlations were then performed between these altered nodal metrics and substance-use-related clinical variables (SUD diagnostic criteria items, etomidate average dose per use, and etomidate use duration), controlling for age, years of education, and mean FD. Statistical significance was assessed using false discovery rate (FDR) correction across all tested correlations.

Statistical significance was set at *p* < 0.05, with Bonferroni correction for multiple comparisons. Partial correlation analyses were then used within the EM group to explore the associations between graph metrics and substance use characteristics as well as clinical variables.

### 2.7 Support Vector Machine Analysis

The graph metrics were used as features to discriminate EM from HC through SVM analysis, implemented using the LIBSVM toolbox version 3.31 (Chih-Jen L, National Taiwan University, Taipei, Taiwan; https://www.csie.ntu.edu.tw/~cjlin/libsvm/) in MATLAB. The initial feature set consisted of global metrics and nodal metrics derived from brain regions showing significant between-group differences. To reduce the risk of overfitting, recursive feature elimination (RFE) was applied within the training data, and features were ranked according to their selection frequency. The top 30% of features were retained for classification. The retained features and their corresponding brain regions/graph metrics are summarized in **Supplementary Table 1**. To address class imbalance, the Synthetic Minority Over-sampling Technique (SMOTE) was applied exclusively within each training fold during the 10-fold cross-validation process. An RBF kernel was used, and internal optimization of the C and γ parameters was performed within the training data. The final accuracy was calculated as the mean accuracy obtained across all testing phases. To evaluate the classifier’s performance, metrics such as accuracy, sensitivity, specificity, and the area under the receiver operating characteristic curve (AUC) were used. Permutation tests with 5000 iterations were conducted to assess the statistical significance of the accuracy.

## 3. Results

### 3.1 Demographics and Clinical Measures

A total of 112 participants were included in the EM group and 59 HC were recruited from the local community. In the data preprocessing stage, 2 healthy controls and 9 individuals with EM were excluded on account of excessive head motion. Accordingly, data from a total of 160 male participants (57 healthy controls and 103 EM patients) were included in the final analysis. Significant differences were observed between the EM and HC groups in terms of age, years of education, and employment rate. No statistically significant differences were found between the groups regarding ethnicity, marital status, only-child status, or proportion of single-parent families. The EM group began using substances at an average age of 18.47 years and had an average etomidate dose of 1.40 grams per use over a duration of 7.08 months. They reported a drug craving score of 41.67 ± 16.11 and had a median of 9 positive diagnostic criteria for SUD. The BIS-11 results showed significantly higher impulsivity in the EM group compared to the HC group (total and all subscale scores, *p* < 0.001) (See Table 1).

**Table 1.** **Demographic information**.

	HC (n = 57)	EM (n = 103)	χ^2^/t	*p*
Male N (%)	57 (100%)	103 (100%)	–	–
Age (years)	21.46 ± 3.40	20.00 ± 4.08	2.235	0.027
Education (years)	14.16 ± 1.60	9.51 ± 2.15	14.253	<0.001
Han nationality N (%)	53 (93.0%)	102 (99.0%)		0.055
Employ N (%)				
Employed	56 (98.2%)	66 (64.1%)	23.655	<0.001
Unemployed	1 (1.8%)	37 (35.9%)		
Marital status				
Unmarried	54 (94.7%)	93 (90.3%)	1.060	0.675
Married	3 (5.3%)	8 (7.8%)		
Divorced	0 (0%)	2 (1.9%)		
Only child N (%)	19 (33.3%)	26 (25.2%)	1.188	0.276
Single-parent family N (%)	7 (12.3%)	25 (24.3%)	3.298	0.069
Initial age of substance use (years)		18.47 ± 3.44		
Etomidate average dose per use (grams)		1.40 ± 1.17		
Etomidate use duration (months)		7.08 ± 6.45		
SUD diagnostic criteria items		9.0 (7.0, 10.0)		
DDQ score		41.67 ± 16.11		
Drug craving		2.73 ± 1.54		
Negative reinforcement		3.30 ± 1.77		
Control		3.61 ± 1.64		
BIS-11 score	70.09 ± 13.61	88.92 ± 17.53	–7.544	<0.001
Motor impulsivity	21.37 ± 5.31	27.04 ± 7.75	–5.462	<0.001
Cognitive impulsivity	24.35 ± 5.01	29.83 ± 7.06	–5.704	<0.001
Non-planning impulsivity	24.37 ± 6.56	32.05 ± 8.17	–6.484	<0.001
Days of abstinence		48.97 ± 44.68		

Note: Continuous data are presented as the mean ± standard deviation (SD) or median (P25, P75), depending on the distribution of the data. Categorical data are presented as a count (percentage). Abbreviations: HC, healthy control; EM, etomidate misuse; SUD, substance use disorder; DDQ, Desire for Drug Questionnaire; BIS-11, Barratt Impulsiveness Scale, version 11.

### 3.2 Resting-State Brain Functional Topology Metrics

**Fig. 1.**
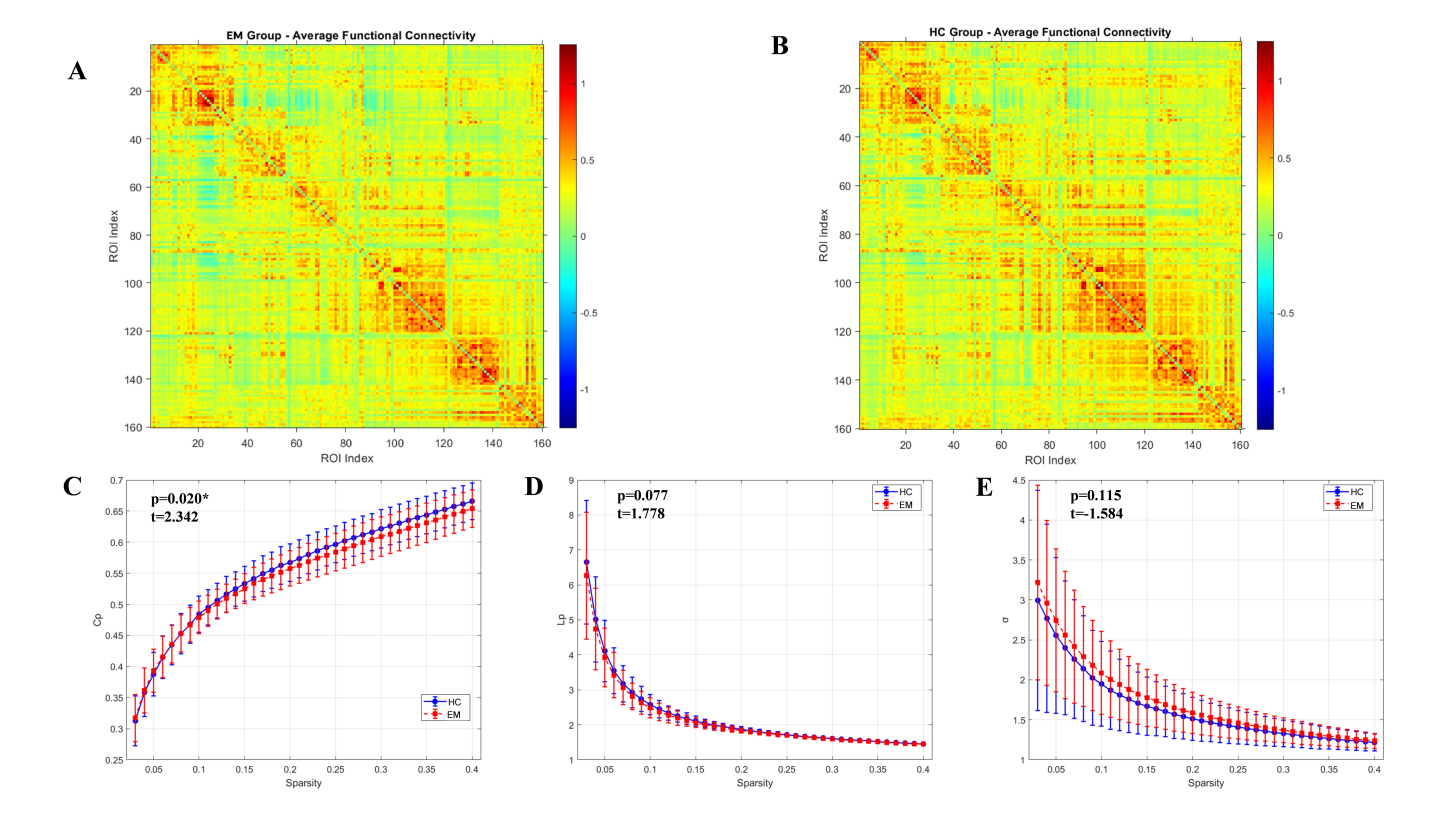
**Global functional connectivity matrices and topological metrics**. (A,B) Group-averaged functional connectivity matrices for the EM group (A) and the HC group (B). The color scale represents the strength of the Pearson correlation coefficients between 160 brain regions. Global graph metrics characteristics of HC and EM (C–E). ROI, regions of interest; C_p_, clustering coefficient; L_p_, shortest path length; σ, small-worldness. *, *p < 0.05.*

Age, years of education, and mean FD were incorporated as covariates in the comparative group analyses. To provide a comprehensive overview of the brain’s functional organization, group-averaged functional connectivity matrices were constructed for both the EM and HC groups (Fig. 1A,B, ROI labels correspond to **Supplementary Table 2**). Compared to HC, EM exhibited significantly reduced clustering coefficient (C_p_, *p* = 0.020) (Fig. 1C,D,E). However, no significant differences were detected in the other global-level topological characteristics, including small-worldness (σ), global efficiency (E_g_), local efficiency (E_loc_), and shortest path length (L_p_).

**Fig. 2.**
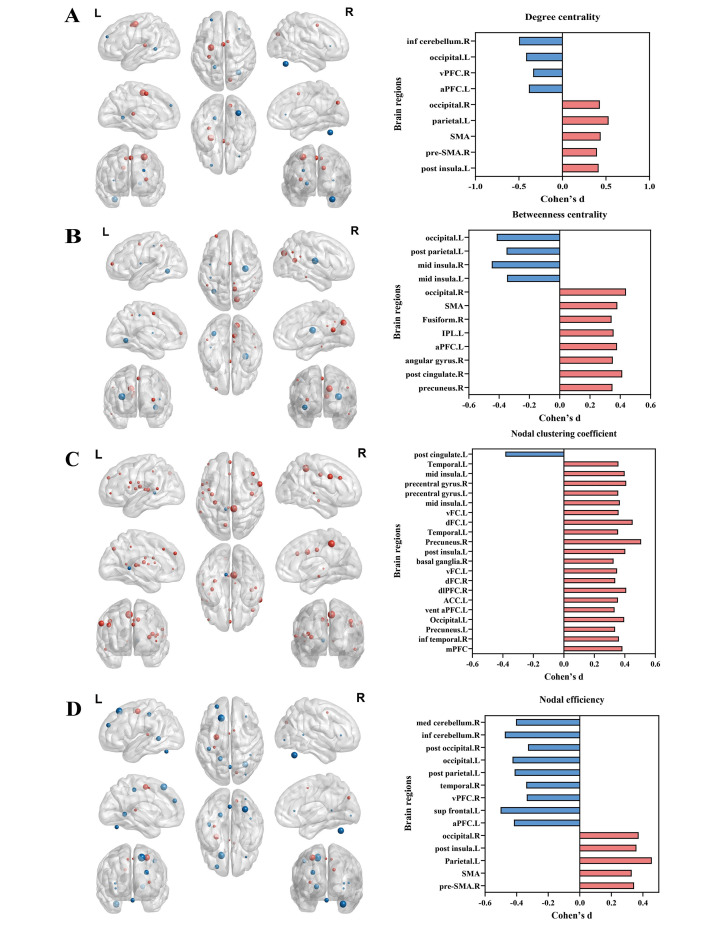
**Nodal metrics between HC and EM groups**. Figures A through D respectively display the degree centrality, betweenness centrality, nodal clustering coefficient, and nodal efficiency values for the HC and EM groups, along with the corresponding Cohen’s d effect sizes. Note: The red nodes indicate areas where HC > EM, and blue nodes represent regions where EM > HC. The size of the nodes corresponds to the effect size. All results were corrected for multiple comparisons using Bonferroni correction (*p* < 0.05).

At the nodal level, we analyzed degree centrality, betweenness centrality, nodal efficiency, nodal local efficiency, shortest path length, and nodal clustering coefficient. Compared to HC, EM showed significant changes in degree centrality at 9 nodes (*p* < 0.05, Bonferroni-corrected), with changes in betweenness centrality observed at 12 nodes. Nodal local efficiency was altered in 13 nodes, and nodal efficiency was altered in 14 nodes (Fig. 2). These altered nodes were distributed across several brain networks, including the default mode network, ventral attention network, dorsal attention network, frontoparietal network, limbic system, sensorimotor network, and visual network. However, group differences in shortest path length did not survive Bonferroni correction for multiple comparisons. Fig. 2 illustrates these findings, showing the changes in betweenness centrality, degree centrality, nodal efficiency, and nodal clustering coefficient across the brain. Detailed information about these results is provided in the **Supplementary Table 3**.

### 3.3 Graph Metrics and Clinical Variables

Partial correlation analyses were conducted to examine associations between altered nodal metrics and substance-use-related clinical variables. At the uncorrected level (*p* < 0.05), 12 node-metric–clinical variable associations were observed, particularly in the insula, where three nodal metrics showed associations with both SUD diagnostic criteria and etomidate average dose per use (see **Supplementary Table 4** for the full list). These results are exploratory and should be interpreted with caution. However, after applying FDR correction, no correlations were observed between graph metrics and SUD diagnostic criteria items, etomidate average dose per use, or etomidate use duration in EM group.

### 3.4 Support Vector Machine Analysis

**Fig. 3.**
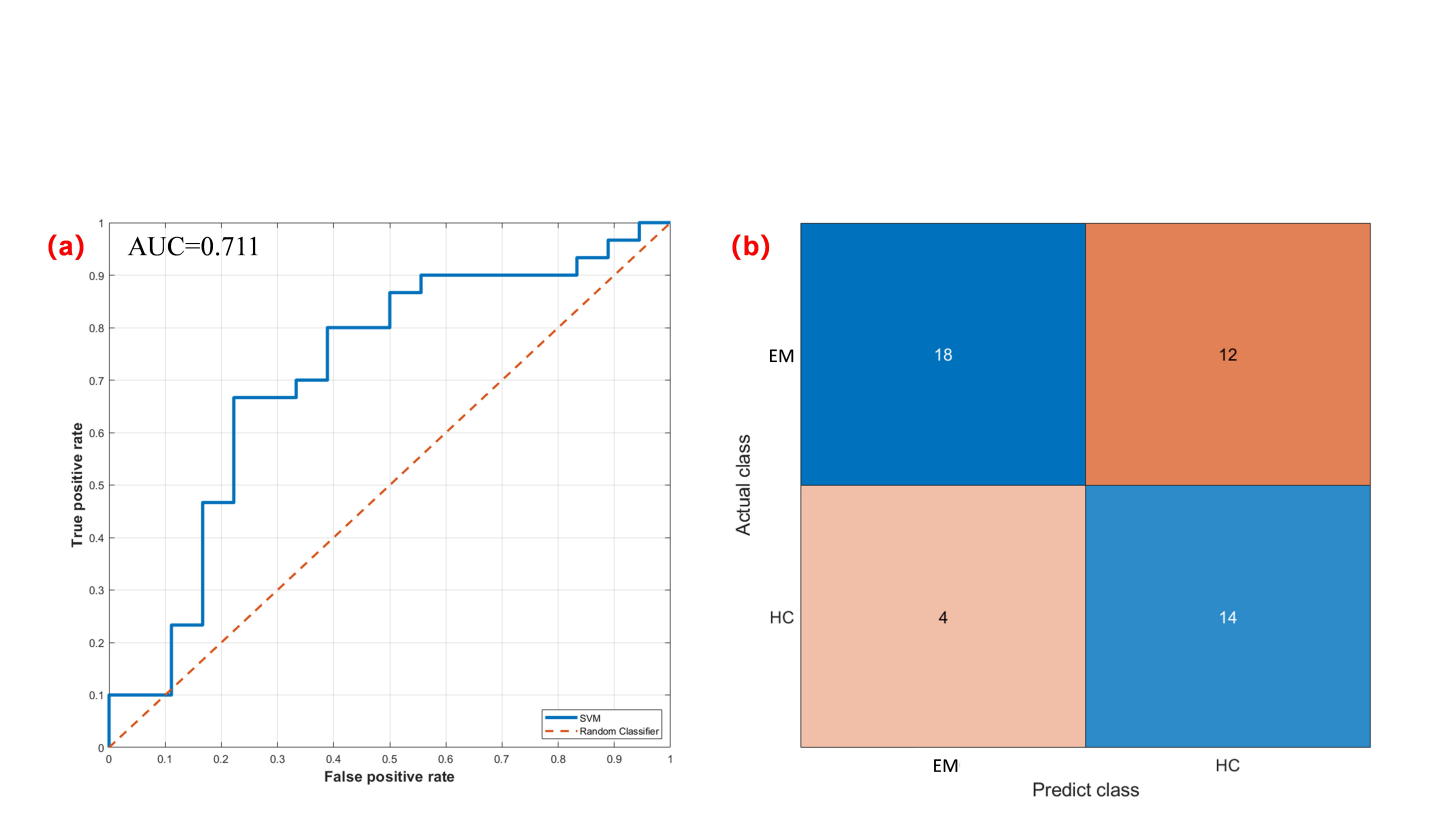
**SVM: Classification of EM and HC based on graph theory metrics**. Part a is receiver operating characteristic curve, while b is the confusion matrix. SVM, support vector machine; AUC, area under the curve.

The results of the SVM classifier using graph theory metrics were as follows: area under the curve (AUC) = 0.711, accuracy = 66.7%, specificity = 77.8%, sensitivity = 60.0%, with a permutation test *p*-value < 0.001 (Fig. 3). Specifically, as detailed in the confusion matrix (Fig. 3b), the model correctly predicted 18 out of 30 individuals who misuse etomidate, and 14 out of 18 healthy controls.

## 4. Discussion

This study is the first to systematically investigate the functional brain network topology in individuals with EM through graph theory-based methods. Our findings reveal that individuals with EM exhibited significantly reduced global clustering coefficients (Cp) at the network level. Furthermore, widespread abnormalities were observed at the nodal level, with significant alterations in key topological metrics, such as degree centrality, betweenness centrality, and local efficiency, across several brain regions. After applying FDR correction, no significant correlations were found between altered nodal metrics and substance-use-related clinical variables. Moreover, a classification model using topological metrics showed a moderate ability to distinguish between EM and HC, suggesting its potential as a diagnostic tool. Collectively, this work provides critical evidence for understanding the neurobiological basis of EM and identifies candidate neuroimaging biomarkers for clinical application.

Regarding local network properties, the EM group exhibited widespread abnormalities, affecting a range of metrics such as degree centrality, betweenness centrality, nodal efficiency, nodal local efficiency and the nodal clustering coefficient. Our findings encompass functional alterations in multiple large-scale brain networks, including the default mode network, dorsal/ventral attention networks, frontoparietal control network, limbic system, sensorimotor network and visual network. These neurofunctional alterations demonstrate that brain network function is disrupted at multiple topological and functional levels in individuals with EM. Such extensive changes in brain network node properties are relatively rare in current SUD research [35,36,37]. Wang documented that alcohol use disorder is associated with reduced global and local network efficiency, accompanied by altered functional connectivity in regions related to multi-sensory modalities. Specifically, the nodal efficiency of the left orbitofrontal cortex (OFC) increased, while that of the right orbitofrontal cortex, right fusiform gyrus, and left insula all decreased [38]. Similarly, Mansoory et al. [39] found that methamphetamine use disorder is associated with significant alterations in resting-state functional connectivity within the default mode network, executive control network. Additionally, stimulant use disorder is predominantly characterized by decreased functional connectivity between the striatum and default mode network, alongside attenuated functional connectivity between the limbic system and prefrontal cortex [40]. In contrast, changes in brain network node properties among EM individuals encompass multiple distinct brain regions and functional networks. These findings may be attributed to the unique neuropharmacological properties of etomidate, which are primarily mediated through the potentiation of GABA_A receptor function [41]. Research has shown that GABA_A receptors are widely expressed across the central nervous system, with particularly enriched expression in brain regions including the cerebral cortex, hippocampus, basal ganglia, and cerebellum [42,43]. This widespread neuroanatomical distribution may contribute to more extensive functional disruption across brain networks in individuals with EM, as opposed to alterations confined to discrete brain regions.

Regarding global network properties, compared to HC, no significant differences were observed in small-worldness (σ), global efficiency (Eg), local efficiency (Eloc), and characteristic path length (Lp). These findings suggest that EM individuals exhibit reduced local clustering and modularity in functional networks, with a preserved capacity for global integration and small-worldness. Cumulative evidence from prior studies has established that long-term SUDs are characterized by more pronounced disruptions in global network topology. For instance, methamphetamine use disorder show reduced global efficiency, increased characteristic path length, and diminished small-worldness, all pointing to compromised global integration capabilities [11,44]. Schweitzer et al. [45] performed network analysis underlying visuospatial working memory performance and showed that the cocaine use disorder group had lower global efficiency than the non-exposed group and a trend-level reduction in local efficiency. Separately, after controlling for functional connectivity differences and network density, individuals with cocaine use disorder showed reduced communication efficiency and small-worldness [46]. In contrast, individuals who misuse etomidate primarily presents a profile of impaired network segregation with relatively preserved global integration. Two key factors may underlie this pattern: first, the mean duration of drug use in this sample (~7 months), in contrast to the multi-year exposure typical of conventional SUDs, suggests an early phase of network alteration that impacts local connectivity while sparing global integration [47,48]. And second, etomidate possesses a distinct pharmacological profile from psychostimulants such as methamphetamine and cocaine: it exerts its primary pharmacodynamic effects via positive allosteric modulation of GABA_A receptors, mediating sedative and anesthetic effects as opposed to the excitatory neurochemical cascades elicited by psychostimulants [15,16,41]. Collectively, these findings indicate that, in the early stage of etomidate exposure represented by the current cohort, functional brain network alterations manifest initially in local network segregation, while global small-world topological properties remain relatively preserved.

Our exploratory analyses identified several uncorrected associations between insular nodal metrics and substance-use characteristics. However, none of these associations survived FDR correction, underscoring the importance of controlling for multiple comparisons in neuroimaging studies. These results should therefore be considered preliminary. The lack of robust correlations after correction may reflect limited statistical power and highlights the need for larger, independent samples to determine whether these trends represent true clinical–neurobiological relationships.

Additionally, SVM analysis was performed to evaluate the ability of topological features to discriminate between EM and HC. The results indicate that machine learning models utilizing graph theory metrics showed moderate discrimination EM from HC, with high specificity (77.8%) and moderate sensitivity (60.0%). While the current accuracy of 66.7% suggests potential utility that requires further validation of EM neural network features, there is significant room for improvement, particularly in enhancing model sensitivity. These results suggest that nodal metrics could serve as preliminary neuroimaging markers for EM, though their clinical utility requires further validation in larger, independent cohorts.

### Strengths and Limitations

Despite being an emerging substance associated with problematic use, EM has received limited attention in neuroimaging research, even though other substance use disorders have been linked to abnormal brain network topology. To our knowledge, this is the first neuroimaging study on EM, making a significant contribution to the field. By identifying widespread changes in brain network nodal properties in individuals with EM, this study provides novel insights into the neurobiological underpinnings of EM and identifies preliminary neuroimaging evidence for the development of future clinical diagnostic and interventional strategies.

Nevertheless, several limitations should be acknowledged. First, the generalizability of our findings is limited by the exclusive focus on males. Future work should include both sexes to determine sex-related influences on network topology in EM. Second, the present study employed graph-theoretic analyses based exclusively on rs-fMRI data. Evidence suggests an association between white matter (WM) abnormalities and addiction [49]. Therefore, future studies should incorporate WM imaging modalities such as diffusion tensor imaging. Third, the relatively small sample size limits statistical power and necessitates validation in larger cohorts. Fourth, there were between-group differences in age and years of education. While these variables were included as covariates in the statistical analyses to mitigate potential biases, such adjustments may still carry the risk of over-correction. Future studies should aim to increase sample sizes and reduce group differences in these variables to better control for confounding and improve the robustness of the findings. Besides, this study utilized only the Dosenbach atlas for brain parcellation. Future studies employing multiple brain atlases will be crucial for assessing the robustness and reproducibility of the current findings. Finally, the cross-sectional nature of our study precludes insights into the temporal evolution of brain network changes in EM. Longitudinal studies are therefore essential to track these developmental trajectories and clarify the neurobiological progression of the disorder.

## 5. Conclusions

This study constitutes the first systematic exploration of the relationship between EM and the topological architecture of functional brain networks. Our findings reveal that individuals with EM exhibit significant abnormalities in global topological properties and more extensive changes in nodal properties across multiple brain regions. In addition, SVM models based on topological features showed moderate discrimination between EM and HC. Together, these findings identify candidate network-level markers of EM that warrant validation in larger and independent samples.

## Data Availability

The datasets used and analyzed during the current study are available from the corresponding author on reasonable request.

## References

[1] Gao T, Jin M, Alibudbud R, Assanangkornchai S, Sun Y, Lu L (2025). Etomidate misuse: a digital era threat to youth and a call for anticipatory control. The Lancet. Psychiatry.

[2] Ministry of Public Security of the People's Republic of China (2024). *Ministry of Public Security of the People's Republic of China. Report on the drug situation in China in 2024*.

[3] Chung YK, Cheung YT, Chan CSY, Wong CC, Fu ACC, Lam YY (2025). Adrenal insufficiency due to etomidate inhalation via electronic cigarettes: three local cases. Hong Kong Medical Journal = Xianggang Yi Xue Za Zhi.

[4] Liu CI, Chen IM, Wang SY, Chen C (2025). Emergence of a New Threat in East Asia: Severe Agitation and Suicide Attempts Linked to Etomidate-Infused E-Cigarettes. Asia-Pacific Psychiatry : Official Journal of the Pacific Rim College of Psychiatrists.

[5] Uhm J, Hong S, Han E (2024). The need to monitor emerging issues in etomidate usage: the misuse or abuse potential. Forensic Science, Medicine, and Pathology.

[6] Yan H, Xiao S, Fu S, Gong J, Qi Z, Chen G (2023). Functional and structural brain abnormalities in substance use disorder: A multimodal meta-analysis of neuroimaging studies. Acta Psychiatrica Scandinavica.

[7] Zeng X, Han X, Zheng D, Jiang P, Yuan Z (2024). Similarity and difference in large-scale functional network alternations between behavioral addictions and substance use disorder: a comparative meta-analysis. Psychological Medicine.

[8] Zilverstand A, Huang AS, Alia-Klein N, Goldstein RZ (2018). Neuroimaging Impaired Response Inhibition and Salience Attribution in Human Drug Addiction: A Systematic Review. Neuron.

[9] Baghernezhad S, Daliri MR (2024). Age-related changes in human brain functional connectivity using graph theory and machine learning techniques in resting-state fMRI data. GeroScience.

[10] Khodadadi Arpanahi S, Hamidpour S, Ghasvarian Jahromi K (2025). Binary and weighted network analysis and its applications to functional connectivity in subjective memory complaints: A resting-state fMRI approach. Ageing Research Reviews.

[11] Cheng P, Li Y, Wang G, Dong H, Liu H, Shen W (2023). Aberrant topology of white matter networks in patients with methamphetamine dependence and its application in support vector machine-based classification. Scientific Reports.

[12] Yang W, Wen X, Du Z, Yang L, Chen Y, Zhang J (2025). Distinct insula subdivisions of resting-state functional connectivity in individuals with opioid and methamphetamine use disorders. Cerebral Cortex (New York, N.Y. : 1991).

[13] Zilverstand A, Parvaz MA, Moeller SJ, Kalayci S, Kundu P, Malaker P (2023). Whole-brain resting-state connectivity underlying impaired inhibitory control during early versus longer-term abstinence in cocaine addiction. Molecular Psychiatry.

[14] Kim DJ, Skosnik PD, Cheng H, Pruce BJ, Brumbaugh MS, Vollmer JM (2011). Structural network topology revealed by white matter tractography in cannabis users: a graph theoretical analysis. Brain Connectivity.

[15] Pence A, Hoyt H, McGrath M, Forman SA, Raines DE (2022). Competitive Interactions Between Propofol and Diazepam: Studies in GABAA Receptors and Zebrafish. The Journal of Pharmacology and Experimental Therapeutics.

[16] Tobias JD, Leder M (2011). Procedural sedation: A review of sedative agents, monitoring, and management of complications. Saudi Journal of Anaesthesia.

[17] Valk BI, Struys MMRF (2021). Etomidate and its Analogs: A Review of Pharmacokinetics and Pharmacodynamics. Clinical Pharmacokinetics.

[18] Akhtar SMM, Saleem SZ, Rizvi SHA, Raja S, Asghar MS (2023). Beyond the surface: analyzing etomidate and propofol as anesthetic agents in electroconvulsive therapy-A systematic review and meta-analysis of seizure duration outcomes. Frontiers in Neurology.

[19] Chen Y, Wu L, Lang B, Zhang W, Chen S (2025). Recent progress in the development of etomidate analogues. Frontiers in Pharmacology.

[20] Hasin DS, O'Brien CP, Auriacombe M, Borges G, Bucholz K, Budney A (2013). DSM-5 criteria for substance use disorders: recommendations and rationale. The American Journal of Psychiatry.

[21] Babcock RL (1994). Analysis of adult age differences on the Raven's Advanced Progressive Matrices Test. Psychology and Aging.

[22] Raven J (2000). The Raven's progressive matrices: change and stability over culture and time. Cognitive Psychology.

[23] Sheehan DV, Lecrubier Y, Sheehan KH, Amorim P, Janavs J, Weiller E (1998). The Mini-International Neuropsychiatric Interview (M.I.N.I.): the development and validation of a structured diagnostic psychiatric interview for DSM-IV and ICD-10. The Journal of Clinical Psychiatry.

[24] Korte KJ, Jaguga F, Kim HH, Stroud RE, Stevenson A, Akena D (2023). Psychometric properties of the mini international neuropsychiatric interview (MINI) psychosis module: a Sub-Saharan Africa cross country comparison. Psychological Medicine.

[25] Franken IHA, Hendriksa VM, van den Brink W (2002). Initial validation of two opiate craving questionnaires the obsessive compulsive drug use scale and the desires for drug questionnaire. Addictive Behaviors.

[26] Li Y, Tian Y, Fan F, Chen J, Fu F, Zhu R (2023). Prevalence, demographics, and clinical correlates of antisocial personality disorder in Chinese methamphetamine patients. The American Journal on Addictions.

[27] Xinyu B (2022). *Reliability and Validity Evaluation of the Impulsivity Scale and Its Relationship with Suicide in Rural Older Adults*.

[28] Xianyun L, Lipeng F, Dong X, Yali Z, Shaojie Y, Yongsheng D (2011). Reliability and Validity of the Chinese Revised Version of the Barratt Impulsiveness Scale in Community and University Samples. *Chinese Mental Health Journal*.

[29] Patton JH, Stanford MS, Barratt ES (1995). Factor structure of the Barratt impulsiveness scale. Journal of Clinical Psychology.

[30] Wang J, Wang X, Xia M, Liao X, Evans A, He Y (2015). GRETNA: a graph theoretical network analysis toolbox for imaging connectomics. Frontiers in Human Neuroscience.

[31] Jenkinson M, Bannister P, Brady M, Smith S (2002). Improved optimization for the robust and accurate linear registration and motion correction of brain images. NeuroImage.

[32] Dosenbach NUF, Nardos B, Cohen AL, Fair DA, Power JD, Church JA (2010). Prediction of individual brain maturity using fMRI. Science (New York, N.Y.).

[33] Watts DJ, Strogatz SH (1998). Collective dynamics of 'small-world' networks. Nature.

[34] Liu Z, Zhang Y, Yan H, Bai L, Dai R, Wei W (2012). Altered topological patterns of brain networks in mild cognitive impairment and Alzheimer's disease: a resting-state fMRI study. Psychiatry Research.

[35] Roland AV, Coelho CAO, Haun HL, Gianessi CA, Lopez MF, D'Ambrosio S (2023). Alcohol Dependence Modifies Brain Networks Activated During Withdrawal and Reaccess: A c-Fos-Based Analysis in Mice. Biological Psychiatry.

[36] Bell RP, Cohen JR, Towe SL, Gadde S, Al-Khalil K, Costello A (2024). Chronic cannabis use associated with subcortical topological reorganization of structural connectivity in adults. Drug and Alcohol Dependence.

[37] Mijangos M, Pacheco L, Bravetti A, González-García N, Padilla P, Velasco-Segura R (2024). Persistent homology reveals robustness loss in inhaled substance abuse rs-fMRI networks. PloS One.

[38] Wang Y, Zhao Y, Nie H, Liu C, Chen J (2018). Disrupted Brain Network Efficiency and Decreased Functional Connectivity in Multi-sensory Modality Regions in Male Patients With Alcohol Use Disorder. Frontiers in Human Neuroscience.

[39] Mansoory MS, Allahverdy A, Behboudi M, Khodamoradi M (2022). Local efficiency analysis of restingstate functional brain network in methamphetamine users. Behavioural Brain Research.

[40] Atanasova N, Todeva-Radneva A, Stoyanova K, Psederska E, Dzhambazova E, Stoyanov D (2026). Distinct subcortical connectivity patterns of opioid and stimulant use disorders: A resting-state fMRI study. Psychiatry Research. Neuroimaging.

[41] Herd MB, Lambert JJ, Belelli D (2014). The general anaesthetic etomidate inhibits the excitability of mouse thalamocortical relay neurons by modulating multiple modes of GABAA receptor-mediated inhibition. The European Journal of Neuroscience.

[42] Heldt SA, Ressler KJ (2007). Forebrain and midbrain distribution of major benzodiazepine-sensitive GABAA receptor subunits in the adult C57 mouse as assessed with in situ hybridization. Neuroscience.

[43] Nutt D (2006). GABAA receptors: subtypes, regional distribution, and function. Journal of Clinical Sleep Medicine : JCSM : Official Publication of the American Academy of Sleep Medicine.

[44] Luo D, He W, Shen D, Tang B, Tao H, Tang Q (2024). Alterations in the brain functional network of abstinent male individuals with methamphetamine use disorder. Cerebral Cortex (New York, N.Y. : 1991).

[45] Schweitzer JB, Riggins T, Liang X, Gallen C, Kurup PK, Ross TJ (2015). Prenatal drug exposure to illicit drugs alters working memory-related brain activity and underlying network properties in adolescence. Neurotoxicology and Teratology.

[46] Wang Z, Suh J, Li Z, Li Y, Franklin T, O'Brien C (2015). A hyper-connected but less efficient small-world network in the substance-dependent brain. Drug and Alcohol Dependence.

[47] Suk JW, Hwang S, Cheong C (2021). Functional and Structural Alteration of Default Mode, Executive Control, and Salience Networks in Alcohol Use Disorder. Frontiers in Psychiatry.

[48] Cui S, Cheng F, Yuan Q, Zhang L, Wang L, Zhang K (2021). Association Between Alexithymia, Social Support, and Duration of Methamphetamine Use Among Male Methamphetamine-Dependent Patients. Frontiers in Psychiatry.

[49] Pando-Naude V, Toxto S, Fernandez-Lozano S, Parsons CE, Alcauter S, Garza-Villarreal EA (2021). Gray and white matter morphology in substance use disorders: a neuroimaging systematic review and meta-analysis. Translational Psychiatry.

