# Supplementary material for: Disrupted Functional Brain Network Topology in Etomidate Misuse
Source: Alpha Psychiatry. 2026 Jun 25;27(3):49872. doi: 10.31083/AP49872 (PMC13339792; doi:10.31083/AP49872)
Supplement: Supplementary file 1 [file 2757-8038-27-3-49872-s1.zip › Supplementary Material.docx]

Supplementary Table 1. Graph-theoretical features retained after RFE and their selection frequencies in the SVM classification analysis.

| **Graph metric** | **Brain region** | **Selection frequency** |
| --- | --- | --- |
| nodal efficiency | inf cerebellum.R | 0.9 |
| nodal clustering coefficient | vent aPFC.L | 0.9 |
| nodal clustering coefficient | basal ganglia.L | 0.9 |
| degree centrality | post insula.L | 0.8 |
| degree centrality | vPFC.R | 0.8 |
| degree centrality | pre-SMA.R | 0.7 |
| nodal local efficiency | mid insula.R | 0.7 |
| nodal local efficiency | dlPFC.R | 0.7 |
| degree centrality | parietal.L | 0.7 |
| betweenness centrality | mPFC | 0.7 |
| betweenness centrality | aPFC.L | 0.7 |
| betweenness centrality | vmPFC.R | 0.7 |
| betweenness centrality | vmPFC.L | 0.6 |
| betweenness centrality | vmPFC.L | 0.6 |
| betweenness centrality | vmPFC.R | 0.6 |
| betweenness centrality | vlPFC.R | 0.6 |
| betweenness centrality | sup frontal.R | 0.6 |
| betweenness centrality | inf temporal.R | 0.6 |
| betweenness centrality | inf temporal.L | 0.5 |
| degree centrality | post cingulate.R | 0.5 |
| nodal efficiency | angular gyrus.R | 0.4 |

Supplementary Table 2. List of ROI labels and corresponding brain regions

| **Labels** | **Regions** | **Hemishpere** | **Subnetwork** | **MNI-coordinates** | | |
| --- | --- | --- | --- | --- | --- | --- |
|  |  |  |  | **x(mm)** | **y(mm)** | **z(mm)** |
| 1 | vmPFC | R | default | 6 | 64 | 3 |
| 2 | mPFC |  | default | 0 | 51 | 32 |
| 3 | aPFC | L | default | -25 | 51 | 27 |
| 4 | vmPFC | R | default | 9 | 51 | 16 |
| 5 | vmPFC | L | default | -6 | 50 | -1 |
| 6 | vmPFC | L | default | -11 | 45 | 17 |
| 7 | vmPFC | R | default | 8 | 42 | -5 |
| 8 | ACC | R | default | 9 | 39 | 20 |
| 9 | vlPFC | R | default | 46 | 39 | -15 |
| 10 | sup frontal | R | default | 23 | 33 | 47 |
| 11 | sup frontal | L | default | -16 | 29 | 54 |
| 12 | inf temporal | R | default | 52 | -15 | -13 |
| 13 | inf temporal | L | default | -59 | -25 | -15 |
| 14 | post cingulate | R | default | 1 | -26 | 31 |
| 15 | fusiform | R | default | 28 | -37 | -15 |
| 16 | precuneus | L | default | -3 | -38 | 45 |
| 17 | post cingulate | L | default | -8 | -41 | 3 |
| 18 | inf temporal | L | default | -61 | -41 | -2 |
| 19 | occipital | L | default | -28 | -42 | -11 |
| 20 | post cingulate | L | default | -5 | -43 | 25 |
| 21 | precuneus | R | default | 9 | -43 | 25 |
| 22 | precuneus | R | default | 5 | -50 | 33 |
| 23 | post cingulate | L | default | -5 | -52 | 17 |
| 24 | post cingulate | R | default | 10 | -55 | 17 |
| 25 | precuneus | L | default | -6 | -56 | 29 |
| 26 | post cingulate | L | default | -11 | -58 | 17 |
| 27 | angular gyrus | R | default | 51 | -59 | 34 |
| 28 | angular gyrus | L | default | -48 | -63 | 35 |
| 29 | precuneus | R | default | 11 | -68 | 42 |
| 30 | IPS | L | default | -36 | -69 | 40 |
| 31 | occipital | L | default | -9 | -72 | 41 |
| 32 | occipital | R | default | 45 | -72 | 29 |
| 33 | occipital | L | default | -2 | -75 | 32 |
| 34 | occipital | L | default | -42 | -76 | 26 |
| 35 | aPFC | R | fronto-parietal | 29 | 57 | 18 |
| 36 | aPFC | L | fronto-parietal | -29 | 57 | 10 |
| 37 | vent aPFC | R | fronto-parietal | 42 | 48 | -3 |
| 38 | vent aPFC | L | fronto-parietal | -43 | 47 | 2 |
| 39 | vlPFC | R | fronto-parietal | 39 | 42 | 16 |
| 40 | dlPFC | R | fronto-parietal | 40 | 36 | 29 |
| 41 | ACC | L | fronto-parietal | -1 | 28 | 40 |
| 42 | dlPFC | R | fronto-parietal | 46 | 28 | 31 |
| 43 | vPFC | L | fronto-parietal | -52 | 28 | 17 |
| 44 | dlPFC | L | fronto-parietal | -44 | 27 | 33 |
| 45 | dFC | R | fronto-parietal | 40 | 17 | 40 |
| 46 | dFC | R | fronto-parietal | 44 | 8 | 34 |
| 47 | dFC | L | fronto-parietal | -42 | 7 | 36 |
| 48 | IPL | L | fronto-parietal | -41 | -40 | 42 |
| 49 | IPL | R | fronto-parietal | 54 | -44 | 43 |
| 50 | post parietal | L | fronto-parietal | -35 | -46 | 48 |
| 51 | IPL | L | fronto-parietal | -48 | -47 | 49 |
| 52 | IPL | L | fronto-parietal | -53 | -50 | 39 |
| 53 | IPL | R | fronto-parietal | 44 | -52 | 47 |
| 54 | IPS | L | fronto-parietal | -32 | -58 | 46 |
| 55 | IPS | R | fronto-parietal | 32 | -59 | 41 |
| 56 | aPFC | R | cingulo-opercular | 27 | 49 | 26 |
| 57 | vPFC | R | cingulo-opercular | 34 | 32 | 7 |
| 58 | ACC | L | cingulo-opercular | -2 | 30 | 27 |
| 59 | vFC | R | cingulo-opercular | 51 | 23 | 8 |
| 60 | ant insula | R | cingulo-opercular | 38 | 21 | -1 |
| 61 | dACC | R | cingulo-opercular | 9 | 20 | 34 |
| 62 | ant insula | L | cingulo-opercular | -36 | 18 | 2 |
| 63 | basal ganglia | L | cingulo-opercular | -6 | 17 | 34 |
| 64 | mFC |  | cingulo-opercular | 0 | 15 | 45 |
| 65 | vFC | L | cingulo-opercular | -46 | 10 | 14 |
| 66 | basal ganglia | L | cingulo-opercular | -20 | 6 | 7 |
| 67 | basal ganglia | R | cingulo-opercular | 14 | 6 | 7 |
| 68 | vFC | L | cingulo-opercular | -48 | 6 | 1 |
| 69 | mid insula | R | cingulo-opercular | 37 | -2 | -3 |
| 70 | thalamus | L | cingulo-opercular | -12 | -3 | 13 |
| 71 | thalamus | L | cingulo-opercular | -12 | -12 | 6 |
| 72 | thalamus | R | cingulo-opercular | 11 | -12 | 6 |
| 73 | mid insula | R | cingulo-opercular | 32 | -12 | 2 |
| 74 | mid insula | L | cingulo-opercular | -30 | -14 | 1 |
| 75 | basal ganglia | R | cingulo-opercular | 11 | -24 | 2 |
| 76 | post insula | L | cingulo-opercular | -30 | -28 | 9 |
| 77 | temporal | R | cingulo-opercular | 51 | -30 | 5 |
| 78 | post cingulate | L | cingulo-opercular | -4 | -31 | -4 |
| 79 | fusiform | R | cingulo-opercular | 54 | -31 | -18 |
| 80 | precuneus | R | cingulo-opercular | 8 | -40 | 50 |
| 81 | parietal | R | cingulo-opercular | 58 | -41 | 20 |
| 82 | temporal | R | cingulo-opercular | 43 | -43 | 8 |
| 83 | parietal | L | cingulo-opercular | -55 | -44 | 30 |
| 84 | sup temporal | R | cingulo-opercular | 42 | -46 | 21 |
| 85 | angular gyrus | L | cingulo-opercular | -41 | -47 | 29 |
| 86 | temporal | L | cingulo-opercular | -59 | -47 | 11 |
| 87 | TPJ | L | cingulo-opercular | -52 | -63 | 15 |
| 88 | frontal | R | sensorimotor | 58 | 11 | 14 |
| 89 | dFC | R | sensorimotor | 60 | 8 | 34 |
| 90 | vFC | L | sensorimotor | -55 | 7 | 23 |
| 91 | pre-SMA | R | sensorimotor | 10 | 5 | 51 |
| 92 | vFC | R | sensorimotor | 43 | 1 | 12 |
| 93 | SMA |  | sensorimotor | 0 | -1 | 52 |
| 94 | frontal | R | sensorimotor | 53 | -3 | 32 |
| 95 | precentral gyrus | R | sensorimotor | 58 | -3 | 17 |
| 96 | mid insula | L | sensorimotor | -42 | -3 | 11 |
| 97 | precentral gyrus | L | sensorimotor | -44 | -6 | 49 |
| 98 | parietal | L | sensorimotor | -26 | -8 | 54 |
| 99 | precentral gyrus | R | sensorimotor | 46 | -8 | 24 |
| 100 | precentral gyrus | L | sensorimotor | -54 | -9 | 23 |
| 101 | precentral gyrus | R | sensorimotor | 44 | -11 | 38 |
| 102 | parietal | L | sensorimotor | -47 | -12 | 36 |
| 103 | mid insula | R | sensorimotor | 33 | -12 | 16 |
| 104 | mid insula | L | sensorimotor | -36 | -12 | 15 |
| 105 | temporal | R | sensorimotor | 59 | -13 | 8 |
| 106 | parietal | L | sensorimotor | -38 | -15 | 59 |
| 107 | parietal | L | sensorimotor | -47 | -18 | 50 |
| 108 | parietal | R | sensorimotor | 46 | -20 | 45 |
| 109 | parietal | L | sensorimotor | -55 | -22 | 38 |
| 110 | precentral gyrus | L | sensorimotor | -54 | -22 | 22 |
| 111 | temporal | L | sensorimotor | -54 | -22 | 9 |
| 112 | parietal | R | sensorimotor | 41 | -23 | 55 |
| 113 | post insula | R | sensorimotor | 42 | -24 | 17 |
| 114 | parietal | R | sensorimotor | 18 | -27 | 62 |
| 115 | parietal | L | sensorimotor | -38 | -27 | 60 |
| 116 | parietal | L | sensorimotor | -24 | -30 | 64 |
| 117 | post parietal | L | sensorimotor | -41 | -31 | 48 |
| 118 | temporal | L | sensorimotor | -41 | -37 | 16 |
| 119 | temporal | L | sensorimotor | -53 | -37 | 13 |
| 120 | sup parietal | R | sensorimotor | 34 | -39 | 65 |
| 121 | occipital | L | occipital | -18 | -50 | 1 |
| 122 | occipital | L | occipital | -34 | -60 | -5 |
| 123 | occipital | R | occipital | 36 | -60 | -8 |
| 124 | temporal | R | occipital | 46 | -62 | 5 |
| 125 | occipital | L | occipital | -44 | -63 | -7 |
| 126 | occipital | R | occipital | 19 | -66 | -1 |
| 127 | occipital | R | occipital | 17 | -68 | 20 |
| 128 | occipital | R | occipital | 39 | -71 | 13 |
| 129 | occipital | R | occipital | 29 | -73 | 29 |
| 130 | occipital | L | occipital | -29 | -75 | 28 |
| 131 | occipital | L | occipital | -16 | -76 | 33 |
| 132 | occipital | R | occipital | 9 | -76 | 14 |
| 133 | occipital | R | occipital | 15 | -77 | 32 |
| 134 | occipital | R | occipital | 20 | -78 | -2 |
| 135 | post occipital | L | occipital | -5 | -80 | 9 |
| 136 | post occipital | R | occipital | 29 | -81 | 14 |
| 137 | post occipital | R | occipital | 33 | -81 | -2 |
| 138 | post occipital | L | occipital | -37 | -83 | -2 |
| 139 | post occipital | L | occipital | -29 | -88 | 8 |
| 140 | post occipital | R | occipital | 13 | -91 | 2 |
| 141 | post occipital | R | occipital | 27 | -91 | 2 |
| 142 | post occipital | L | occipital | -4 | -94 | 12 |
| 143 | lat cerebellum | L | cerebellum | -28 | -44 | -25 |
| 144 | lat cerebellum | L | cerebellum | -24 | -54 | -21 |
| 145 | inf cerebellum | L | cerebellum | -37 | -54 | -37 |
| 146 | lat cerebellum | L | cerebellum | -34 | -57 | -24 |
| 147 | med cerebellum | L | cerebellum | -6 | -60 | -15 |
| 148 | inf cerebellum | L | cerebellum | -25 | -60 | -34 |
| 149 | inf cerebellum | R | cerebellum | 32 | -61 | -31 |
| 150 | med cerebellum | L | cerebellum | -16 | -64 | -21 |
| 151 | lat cerebellum | R | cerebellum | 21 | -64 | -22 |
| 152 | med cerebellum | R | cerebellum | 1 | -66 | -24 |
| 153 | inf cerebellum | L | cerebellum | -34 | -67 | -29 |
| 154 | med cerebellum | L | cerebellum | -11 | -72 | -14 |
| 155 | inf cerebellum | R | cerebellum | 33 | -73 | -30 |
| 156 | med cerebellum | R | cerebellum | 5 | -75 | -11 |
| 157 | med cerebellum | R | cerebellum | 14 | -75 | -21 |
| 158 | inf cerebellum | L | cerebellum | -21 | -79 | -33 |
| 159 | inf cerebellum | L | cerebellum | -6 | -79 | -33 |
| 160 | inf cerebellum | R | cerebellum | 18 | -81 | -33 |

Supplementary Table 3. Abnormal node network characteristics between the HC and EM groups

| **Nodal metrics** | **Brain regions** | ***t*** | ***p*** | **Cohen’s d** |
| --- | --- | --- | --- | --- |
| Degree centrality |  |  |  |  |
|  | aPFC.L | -2.336 | 0.020 | -0.386 |
|  | vPFC.R | -2.044 | 0.043 | -0.337 |
|  | post insula.L | 2.520 | 0.013 | 0.416 |
|  | pre-SMA.R | 2.403 | 0.017 | 0.397 |
|  | SMA | 2.668 | 0.008 | 0.440 |
|  | parietal.L | 3.216 | 0.002 | 0.531 |
|  | occipital.L | -2.525 | 0.013 | -0.417 |
|  | occipital.R | 2.608 | 0.010 | 0.430 |
|  | inf cerebellum.R | -3.023 | 0.003 | -0.499 |
| Betweenness centrality |  |  |  |  |
|  | precuneus.R | 2.116 | 0.036 | 0.349 |
|  | post cingulate.R | 2.500 | 0.014 | 0.413 |
|  | angular gyrus.R | 2.124 | 0.035 | 0.351 |
|  | aPFC.L | 2.298 | 0.023 | 0.379 |
|  | IPL.L | 2.159 | 0.032 | 0.356 |
|  | Fusiform.R | 2.075 | 0.040 | 0.343 |
|  | SMA | 2.310 | 0.022 | 0.381 |
|  | mid insula.L | -2.110 | 0.037 | -0.348 |
|  | mid insula.R | -2.723 | 0.007 | -0.449 |
|  | post parietal.L | -2.125 | 0.035 | -0.351 |
|  | occipital.L | -2.521 | 0.013 | -0.416 |
|  | occipital.R | 2.651 | 0.009 | 0.438 |
| Nodal efficiency |  |  |  |  |
|  | aPFC.L | -2.529 | 0.012 | -0.417 |
|  | sup frontal.L | -3.043 | 0.003 | -0.502 |
|  | vPFC.R | -2.036 | 0.043 | -0.336 |
|  | post insula.L | 2.178 | 0.031 | 0.359 |
|  | temporal.R | -2.054 | 0.042 | -0.339 |
|  | pre-SMA.R | 2.085 | 0.039 | 0.344 |
|  | SMA | 1.992 | 0.048 | 0.329 |
|  | Parietal.L | 2.763 | 0.006 | 0.456 |
|  | post parietal.L | -2.499 | 0.014 | -0.413 |
|  | occipital.L | -2.583 | 0.011 | -0.426 |
|  | occipital.R | 2.260 | 0.025 | 0.373 |
|  | post occipital.R | -1.989 | 0.048 | -0.328 |
|  | inf cerebellum.R | -2.877 | 0.005 | -0.475 |
|  | med cerebellum.R | -2.449 | 0.015 | -0.404 |
| Nodal clustering coefficient |  |  |  |  |
|  | mPFC | 2.324 | 0.021 | 0.384 |
|  | inf temporal.R | 2.188 | 0.030 | 0.361 |
|  | Precuneus.L | 2.036 | 0.043 | 0.336 |
|  | post cingulate.L | -2.324 | 0.021 | -0.384 |
|  | Occipital.L | 2.410 | 0.018 | 0.396 |
|  | vent aPFC.L | 2.017 | 0.045 | 0.333 |
|  | ACC.L | 2.151 | 0.033 | 0.355 |
|  | dlPFC.R | 2.477 | 0.014 | 0.409 |
|  | dFC.R | 2.040 | 0.043 | 0.337 |
|  | vFC.L | 2.116 | 0.036 | 0.349 |
|  | basal ganglia.R | 1.978 | 0.049 | 0.326 |
|  | post insula.L | 2.440 | 0.016 | 0.403 |
|  | Precuneus.R | 3.070 | 0.003 | 0.507 |
|  | Temporal.L | 2.156 | 0.033 | 0.356 |
|  | dFC.L | 2.734 | 0.007 | 0.451 |
|  | vFC.L | 2.172 | 0.031 | 0.359 |
|  | mid insula.L | 2.220 | 0.028 | 0.367 |
|  | precentral gyrus.L | 2.160 | 0.032 | 0.357 |
|  | precentral gyrus.R | 2.475 | 0.014 | 0.409 |
|  | mid insula.L | 2.416 | 0.017 | 0.399 |
|  | Temporal.L | 2.169 | 0.032 | 0.358 |
| Nodal local efficiency |  |  |  |  |
|  | post cingulate.L | -2.959 | 0.004 | -0.488 |
|  | dlPFC.R | 2.160 | 0.032 | 0.357 |
|  | post insula.L | -2.171 | 0.031 | -0.358 |
|  | Precuneus.R | 2.411 | 0.017 | 0.398 |
|  | dFC.R | 2.096 | 0.038 | 0.346 |
|  | precentral gyrus.L | 2.013 | 0.046 | 0.332 |
|  | precentral gyrus.R | 2.043 | 0.043 | 0.337 |
|  | Temporal.L | 2.563 | 0.011 | 0.423 |
|  | vFC.R | 2.326 | 0.021 | 0.384 |
|  | Temporal.L | 2.521 | 0.013 | 0.416 |
|  | inf cerebellum.L | -1.978 | 0.050 | -0.327 |
|  | inf cerebellum.R | -2.239 | 0.027 | -0.370 |
|  | inf cerebellum.L | -2.226 | 0.027 | -0.367 |

Supplementary Table 4. Exploratory correlation analysis between nodal metrics and clinical variables in EM group

| **Clinical Variable** | **Brain Region** | **Metric** | **r** | **p**  **(Uncorrected)** | **p**  **(FDR corrected)** |
| --- | --- | --- | --- | --- | --- |
| **Avg. Dose** |  |  |  |  |  |
|  | SMA | betweenness centrality | 0.195 | 0.047 | 0.844 |
|  | inf cerebellum.R | degree centrality | 0.247 | 0.012 | 0.499 |
|  | temporal.R | nodal efficiency | 0.247 | 0.012 | 0.499 |
|  | mid insula.L | betweenness centrality | 0.260 | 0.008 | 0.499 |
|  | angular gyrus.R | betweenness centrality | -0.262 | 0.007 | 0.499 |
|  | fusiform.R | betweenness centrality | -0.220 | 0.026 | 0.589 |
| **Duration** |  |  |  |  |  |
|  | med cerebellum.R | nodal efficiency | -0.194 | 0.049 | 0.844 |
|  | vFC.R | nodal local efficiency | -0.251 | 0.011 | 0.499 |
| **SUD criteria** |  |  |  |  |  |
|  | post insula.L | degree centrality | -0.234 | 0.017 | 0.512 |
|  | post insula.L | nodal efficiency | -0.237 | 0.016 | 0.512 |
|  | mid insula.R | betweenness centrality | 0.198 | 0.044 | 0.844 |
|  | sup frontal.L | nodal efficiency | 0.226 | 0.022 | 0.560 |

Note: Avg. Dose, Etomidate average dose per use (grams); Duration, Etomidate use duration (months); SUD criteria, SUD diagnostic criteria items.


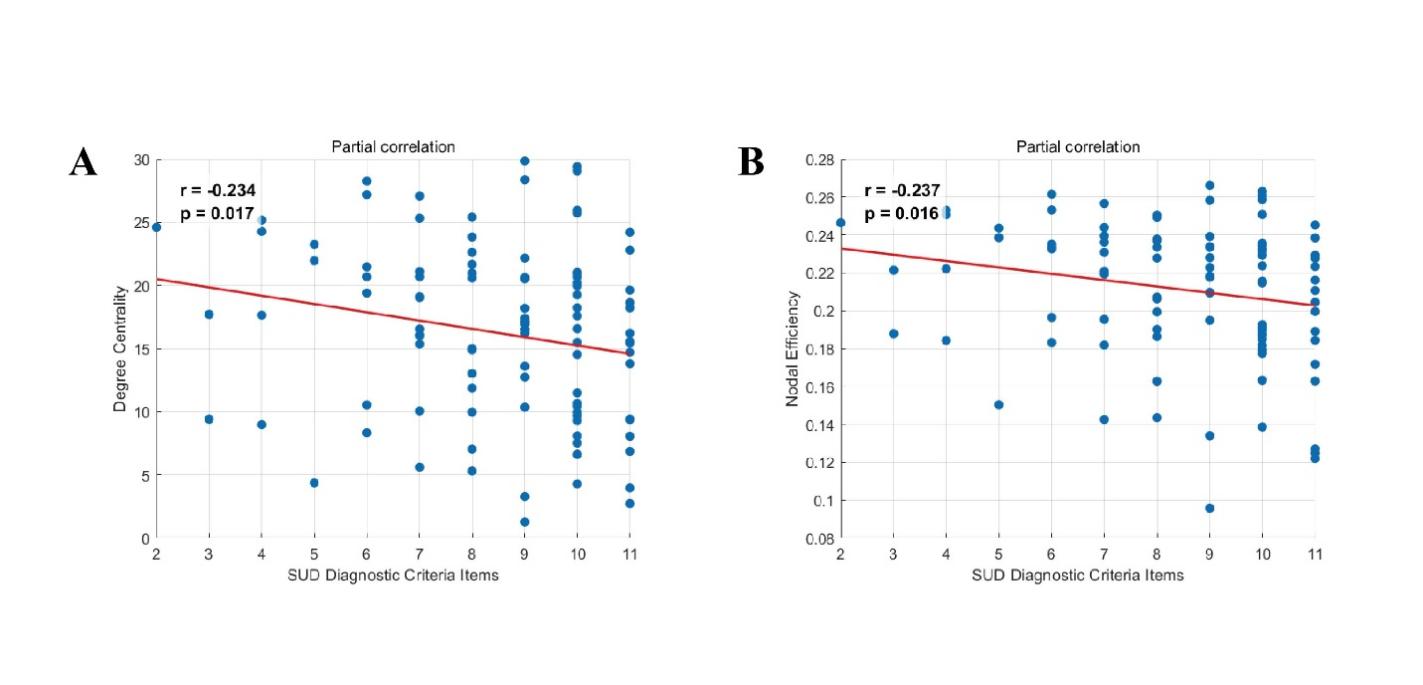


Supplementary Fig. 1. Results of partial correlation analysis among degree centrality, nodal efficiency and SUD diagnostic criteria items (uncorrected).
